# Clinical Lectures on the Principles and Practice of Medicine

**Published:** 1860-05

**Authors:** 


Art. X.—*Clinical Lectures on the Principles and Practice of Medicine.* By John Hughes Bennett, M.D., F.R.S.E., Professor of the Institutes of Medicine, and Senior Professor of Clinical Medicine in the University of Edinburgh, etc. etc. etc. 8vo., pp. 952. New York: S. S. & W. Wood, 1860.

The medical profession of the United States should consider itself deeply indebted to the Messrs. Wood, of New York, for the elegant manner in which they have issued this beautiful and costly work of the Edinburgh professor. The whole plan has been executed in such a masterly manner, the engravings have been so well done, the paper is so white and smooth, and the typography so clear and distinct, as to render it extremely difficult, if not impossible, to distinguish any difference between the English and American editions.

Of the intrinsic value of Professor Bennett's treatise we spoke so fully, on the appearance of the second edition, as to render it unnecessary now to do anything more than to give it a passing notice. Considered all in all, it is beyond question one of the most important, as it is certainly one of the most original, contributions to practical medicine that have been made during the present century, and we can therefore unhesitatingly recommend it to the attentive perusal of every student and practitioner of the healing art, as well calculated to advance their knowledge of the nature and treatment of disease. Composed in a style of unusual beauty and elegance, it abounds in the most valuable pathological and practical suggestions, serving as useful guides at the bedside; it is written in the true spirit of philosophy, and is a perfect model of purity, simplicity, and harmony of language, at the same time that it exhibits a perfect familiarity with all the great improvements of the age, and a masterly application of the microscope and chemical tests to clinical medicine. The present edition, a reprint from the third Edinburgh, has been extended by the addition of fifty pages, and is illustrated by five hundred engravings on wood, executed, as already stated, in the highest style of the art, and of the greatest value in elucidating the text.

Considering the extraordinary merits of Professor Bennett's work, it is not surprising that he should be one of the great pillars of the Edinburgh school, which has never, since the days of Cullen, shone with such a steady and genuine lustre as it does at the present moment under the light of a Simpson, a Syme, a Christison, a Miller, and the author of the Clinical Lectures on the Principles and Practice of Medicine.

